# Clinical simulation training for first responders in pediatric emergencies with family interaction: a scoping review

**DOI:** 10.1590/0034-7167-2024-0218

**Published:** 2025-01-13

**Authors:** Karine Silva Fogaça, Gabriella Figueiredo Marti, Andréia Insabralde de Queiroz Cardoso, Rodrigo Guimarães dos Santos Almeida, Fernanda Ribeiro Baptista Marques, Maria Angélica Marcheti

**Affiliations:** I Universidade Federal de Mato Grosso do Sul. Campo Grande, Mato Grosso do Sul, Brazil

**Keywords:** Emergency Responders, Health Personnel, Simulation Training, Child, Family Nursing, Socorristas, Personal de Salud, Entrenamiento Simulado, Niño, Enfermería de la Familia

## Abstract

**Objective:**

To map studies on clinical simulation training directed at first responders during pediatric emergencies, focusing on interaction with families.

**Methods:**

A scoping review based on the guidelines of the JBI Manual for Evidence Syntheses and reported according to the PRISMA-ScR checklist, covering eight databases and gray literature, without time or language restrictions.

**Results:**

The ten selected studies indicated that most publications were from the United States. Simulations were predominantly conducted in hospital settings, with only one study associated with the pre-hospital context. The main designs used involved pediatric resuscitation scenarios and high-fidelity simulated environments.

**Conclusion:**

Simulation training is effective, increasing professionals’ confidence and improving communication with families. However, the concentration in developed countries and hospital settings highlights the need for training in other settings, such as pre-hospital care, to integrate technical and family-centered approaches.

## RESUMEN

**Objetivo:**

Mapear estudios sobre entrenamientos de simulación clínica dirigidos a los socorristas durante emergencias pediátricas, con un enfoque en la interacción con las familias.

**Métodos:**

Revisión de alcance basada en las directrices del Manual de Síntesis de Evidencia de JBI y reportada según la lista de verificación PRISMA-ScR, cubriendo ocho bases de datos y literatura gris, sin restricciones de tiempo o idioma.

**Resultados:**

Los diez estudios seleccionados indicaron que la mayoría de las publicaciones eran de los Estados Unidos. Las simulaciones se realizaron predominantemente en entornos hospitalarios, con solo un estudio asociado al contexto prehospitalario. Los principales diseños utilizados involucraron escenarios de reanimación pediátrica y entornos simulados de alta fidelidad.

**Conclusión:**

El entrenamiento en simulación es efectivo, aumenta la confianza de los profesionales y mejora la comunicación con las familias. Sin embargo, la concentración en países desarrollados y entornos hospitalarios resalta la necesidad de capacitación en otros contextos, como la atención prehospitalaria, para integrar enfoques técnicos y centrados en la familia.

## INTRODUCTION

Professionals who include the family in the care plan, regardless of health status, adhere to the principles of Patient and Family Centered Care (PFCC)^(1)^. This care model advocates for clear and objective assistance with integrated planning, provision, and evaluation, aimed at the well-being of individuals and families^(2)^. PFCC is ideal for professionals working in patient and family care across various health services^(3)^.

The family is considered an essential part of the health-disease process of its members^(4)^. During emergency care, the presence alongside the patient, known as bedside presence, is valued^(5-8)^. Studies highlight the benefits of family involvement in pediatric care, including safety, reduced anxiety, emotional support, enhanced understanding, and satisfaction with the care. Additionally, family presence at the time of death facilitates the grieving process^(7-9)^.

The philosophy of PFCC is historically linked to the care of children and the hospital context, primarily because it originated in pediatric inpatient units^(10)^. It is important to note that in emergency conditions, families are vulnerable, under stress, and suffering. At home, family members are usually present from the onset of the first severe symptoms until the arrival of the first responders. In the dynamic context of pre-hospital care (PHC), there are limitations to family presence. Healthcare professionals seek to interact with the family primarily to gather information about the patient; however, feedback is often not communicated clearly. In this scenario, the technical approach becomes crucial in delivering care, and the urgency of tasks directly impacts its efficacy^(11)^.

Pediatric emergency situations require special attention from healthcare professionals due to the biological and psychological peculiarities of children, necessitating specialized resources^(3)^. Professionals must be up-to-date on assessment and treatment standards and familiar with pediatric devices to avoid adversities^(12)^. Consequently, professional training courses often focus on technical aspects, which limits the development of communication skills and empathy with the Family^(13)^.

Implementing the family-centered model in emergency care can be challenging^(14)^, but allowing families to choose to remain by the patient’s side is one way to introduce it^(15-17)^. For this, it is necessary to enhance the qualifications and training of first responders, promoting continuous learning and a reflective approach, aimed at comprehensive and quality care^(18)^.

For the training of these professionals, the use of simulated scenarios has proven effective, reducing errors and promoting safety and quality in learning^(19)^. Clinical simulation is a motivating approach that improves adherence and reduces dropout in educational processes, as well as assessing technical and non-technical competencies such as communication and professionalism^(20,21)^, showing positive results in the enhancement and satisfaction of healthcare professionals^(22)^. However, there are still few training programs in pediatric emergencies focusing on family-centered care.

For this study, preliminary searches were conducted in the JBI Evidence Synthesis, PROSPERO Systematic Review, Open Science Framework (OSF), and Cochrane Database of Systematic Reviews (CDSR). These searches did not identify any completed or ongoing systematic or scope reviews related to the topic.

In light of the above, it is crucial to recognize actions focused on family interaction during clinical simulation training for first responders in pediatric emergencies. Such analysis can foster professional training initiatives and assist in the creation of institutional norms that ensure and encourage family presence during pediatric emergency care.

## OBJECTIVE

To map studies on clinical simulation training directed at first responders during pediatric emergencies, with a focus on family interaction.

## METHODS

### Ethical Aspects

No review by a Human Research Ethics Committee was necessary, as the material used is publicly available and did not involve human subjects.

### Study Type

This is a scoping review, structured according to the guidelines of the JBI Manual for Evidence Syntheses^(23)^. This technique is ideal for surveying emerging evidence, aiming to develop a “conceptual map,” provide a comprehensive overview of a question, and identify gaps in the research knowledge base, thereby guiding the need for further investigations^(24,25)^. The five stages recommended by JBI were adopted to conduct the steps of this scoping review: 1) Identification of the research question; 2) Search for relevant studies; 3) Selection of studies; 4) Mapping of data; 5) Grouping, summarizing, and presenting the results.

For reporting this review, the checklist according to the recommendations of the Preferred Reporting Items for Systematic Reviews and Meta-Analyses extension for Scoping Reviews (PRISMA-ScR)^(26)^ was utilized.

### Methodological Procedure

The objectives, inclusion criteria, and methods for this scoping review were predefined and documented in a scoping review protocol following JBI guidelines^(23)^. The protocol was registered on the Open Science Framework (OSF) platform under the DOI: ^https://doi.org/10.17605/OSF.IO/YR9QA(27)^.

The study objective, guiding question, and search strategy were defined according to the PCC mnemonic^(23)^, as detailed in Chart 1. The guiding question developed for this study was: How has the process of family interaction been addressed in clinical simulation training for first responders in the pediatric emergency context?

**Chart 1:** Mnemonic and descriptors used in the searches, 2024

Mnemonic	Descriptors	DeCS/MeSH/EMTREE
**Population:** First Responders	Emergency Responders Health Personnel	*Socorristas* Emergency Responders Emergency First Responder First Responder *Pessoal de Saúde* Health Personnel Health Care Provider Healthcare Provider Healthcare Worker Health Care Professional
**Concept:** Family interaction in clinical simulation training	Simulation Training	*Treinamento por Simulação* Simulation Training Interactive Learning
**Context:** Pediatric emergency context.	Child	*Criança* Child Children

The inclusion criteria selected evidence from studies that employed clinical simulation training as an educational strategy for healthcare professionals or first responders in the context of pediatric emergencies. Considered were complete articles, society documents, manuals, guidelines, and protocols, government documents, theses, and dissertations available in full that addressed the theme. Furthermore, the reference lists of publications were reviewed to ensure all relevant studies related to the topic of interest were identified. It is important to note that there was no temporal cut-off or language limitation. Excluded from the study were monographs, reviews, editorials, letters to the editor, expert opinions, abstracts, conference proceedings, and correspondences.

### Search Strategy

The search strategy was organized by two researchers and validated by another researcher experienced in this process. Based on the research question, controlled and indexed descriptors from the Medical Subject Headings (MeSH), EMBASE Subject Headings (EMTREE), CINAHL Headings, and Health Sciences Descriptors (DeCS) were selected. Keywords were chosen based on suggestions from controlled vocabularies and thorough preliminary reading on the theme. The combination of these descriptors utilized the Boolean operators OR and AND, according to the specifics of each database, as shown in Chart 2.

**Chart 2:** Database search strategies, 2024

Database	Search strategies
*Embase* (*Elsevier*)	*(‘rescue personnel’/exp OR ‘rescue personnel’ OR ‘emergency responders’ OR ‘emergency first responder’ OR ‘first responder’ OR ‘health care personnel’/exp OR ‘health care personnel’ OR ‘health personnel’ OR ‘health care provider’ OR ‘healthcare provider’ OR ‘healthcare worker’ OR ‘health care professional’) AND (‘simulation training’/exp OR ‘simulation training’ OR ‘interactive learning’/exp OR ‘interactive learning’) AND (‘child’/exp OR ‘child’ OR ‘children’) AND [embase]/lim*
Biblioteca Virtual em Saúde/BVS	*((emergency responders) OR (emergency first responder) OR (first responder)) OR ((health personnel) OR (health care provider) OR (healthcare provider) OR (healthcare worker) OR (health care professional)) AND ((simulation training) OR (interactive learning)) AND ((child) OR (children))*
MEDLINE Pubmed	*(“Emergency Responders”[MeSH Terms] OR “Emergency First Responder”[Title/Abstract] OR “First Responder”[Title/Abstract] OR (“Health Personnel”[MeSH Terms] OR “Health Care Provider”[Title/Abstract] OR “Healthcare Provider”[Title/Abstract] OR “Healthcare Worker”[Title/Abstract] OR “Health Care Professional”[Title/Abstract])) AND (“Simulation Training”[MeSH Terms] OR “Interactive Learning”[Title/Abstract]) AND (“Child”[MeSH Terms] OR “Children”[Title/Abstract])*
*Web of Science* *WOS*	*(((ALL=(“Emergency Responders” OR “Emergency First Responder” OR “First Responder”)) OR ALL=(“Health Personnel” OR “Health Care Provider” OR “Healthcare Provider” OR “Healthcare Worker” OR “Health Care Professional”)) AND ALL=(“Simulation Training” OR “Interactive Learning”)) AND ALL=(“Child” OR “Children”)*
*Web of Science* *WOS*	*(((ALL=(“Emergency Responders” OR “Emergency First Responder” OR “First Responder”)) OR ALL=(“Health Personnel” OR “Health Care Provider” OR “Healthcare Provider” OR “Healthcare Worker” OR “Health Care Professional”)) AND ALL=(“Simulation Training” OR “Interactive Learning”)) AND ALL=(“Child” OR “Children”)*
*Scopus (Elsevier)*,	*(ALL(‘emergency AND responders’ OR ‘emergency AND first AND responder’ OR ‘first AND responder’) AND ALL(‘simulation AND training’ OR ‘interactive AND learning’) AND ALL(‘child’ OR ‘children’))*
*Cochrane Library*	*((emergency responders) OR (emergency first responder) OR (first responder)):ti,ab,kw OR ((health personnel) OR (health care provider) OR (healthcare provider) OR (healthcare worker) OR (health care professional)):ti,ab,kw AND ((simulation training) OR (interactive learning)):ti,ab,kw AND ((child) OR (children)):ti,ab,kw*
CINAHL EBSCOhost *with Full Text*	*( MH ”Emergency Responders” OR “Emergency Responders” OR “Emergency First Responder” OR “First Responder” ) OR ( MH ”Health Personnel” OR “Health Personnel” OR “Health Care Provider” OR “Healthcare Provider” OR “Healthcare Worker” OR “Health Care Professional” ) AND ( MH “Simulation” OR “Simulation Training” OR “Interactive Learning” ) AND ( MH “Chid” OR “Child” OR “Children” )*
*ScienceDirect*	*{emergency responders} or {health personnel} and {simulation training} and {child}*

A preliminary search was conducted in the National Library of Medicine (MEDLINE/PubMed) database to verify the feasibility of the research. Keywords were defined using MeSH. Searches for publications occurred between January and March 2024, through the proxy access of the Federal University of Mato Grosso do Sul (UFMS), via the journal portal of the Coordination for the Improvement of Higher Education Personnel (CAPES), through the Federated Academic Community (CAFe).

The databases consulted included: Embase (Elsevier), Virtual Health Library (BVS), National Library of Medicine (MEDLINE/PubMed), Web of Science Core Collection (Clarivate Analytics), Scopus (Elsevier), Cochrane Library, CINAHL EBSCOhost with Full Text, and ScienceDirect.

Additionally, a search was conducted in non-indexed sources, known as grey literature, using the same keywords combined with the Boolean operators OR and AND in English and their Portuguese equivalents. The sources consulted included the CAPES Thesis and Dissertation Catalog, the Digital Library of Theses and Dissertations (BDTD), the International Open Access Thesis and Dissertation Bank (OATD), Academic Archive Online (DIVA), the Open Access Scientific Repository of Portugal (RCAAP), and the Open Grey Information System.

The studies were exported to the Mendeley reference manager for identification and removal of duplicates. For selection and evaluation according to the inclusion criteria, the articles were transferred to the Rayyan software^(28)^. The preliminary screening of data was conducted through independent reading of the titles and abstracts by two reviewers. There was no disagreement between the reviewers, thus eliminating the need for a third researcher or to contact the primary authors regarding the data.

The refinement of the studies was based on the eligibility criteria. Articles that contained the three elements of the PCC mnemonic^(23)^ associated with the family presence approach were included, with no limits on language or publication date. Studies that did not address the guiding research question or did not include family presence in the emergency context were excluded.

The studies that comprised the final sample for analysis were then read in full, with data collection carried out on a structured spreadsheet in Microsoft Excel, containing the following analysis variables: citation, publication year, origin, language, design, population, context, educational intervention, simulation training, study objectives, results, and limitations.

The data were described and presented through figures and explanatory tables, with a descriptive analysis of the findings.

## RESULTS

**Figure 1:**
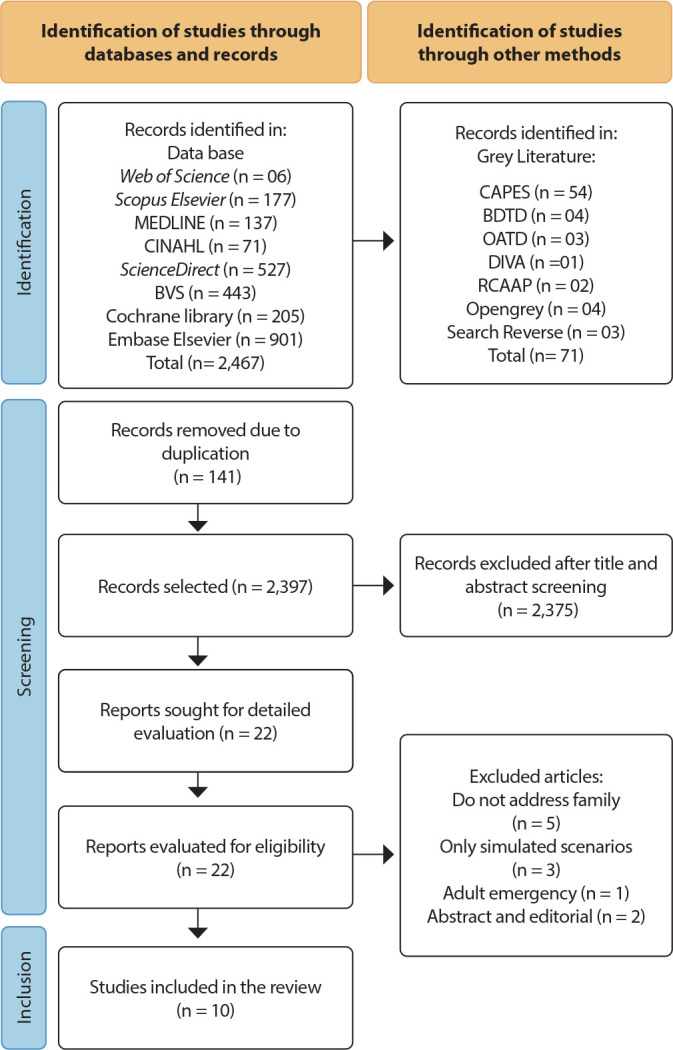
PRISMA-ScR Flowchart for the Identification and Selection of Studies in Databases, 2024

Initially, 2,538 works were identified, with 2,467 found in databases and 71 from other sources. After removing 141 duplicates, 2,397 records remained. In the first selection, which involved reading titles and abstracts, 2,375 records were excluded for not meeting the inclusion criteria, resulting in 22 studies for analysis. In a subsequent screening, five studies were excluded for not addressing family interaction, three for diverging from the concept adopted in this review, one for presenting a different context than defined, and two for not being available in full. Ultimately, the review consisted of 10 publications obtained from the databases, as demonstrated in the PRISMA-ScR flowchart^(26)^ (Figure 1).

The publication period for the eligible studies ranged from 2010 to 2024, without a defined temporal boundary, with the highest occurrence in 2022 (40%). The studies predominantly originated from the United States (60%), followed by Ireland, Canada, Switzerland, and Australia (10% each). The majority of the studies (80%) came from the National Library of Medicine (MEDLINE/PubMed).

The interventions involving clinical simulations primarily consisted of multiprofessional trainings with doctors, nurses, and physical therapists (60%). Nurses in exclusive training accounted for a significant share, at 30%. It is important to note the integration of nurses with the multiprofessional team, demonstrating their frequent involvement in clinical simulation training with family presence during pediatric emergencies (90%).

**Figure 2:**
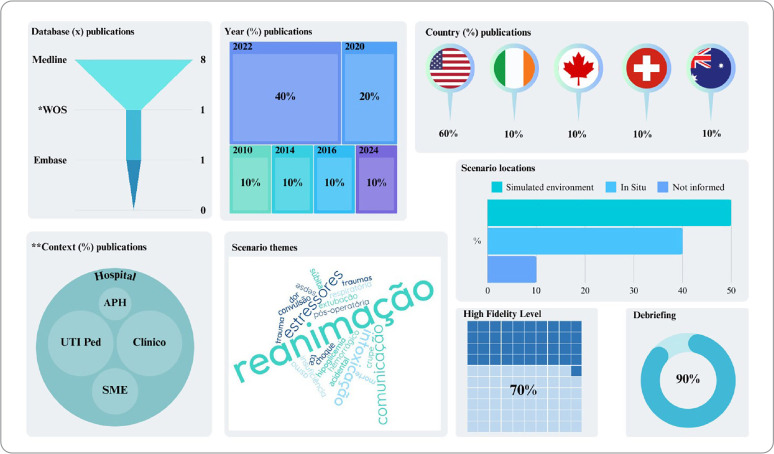
Outcomes and design of clinical simulation trainings with a family approach, 2024

All studies were conducted in hospitals, with 10% also associated with other settings, but none exclusively in pre-hospital care scenarios. Regarding the design of the simulated scenarios conducted, the studies showed a predominance of themes related to pediatric resuscitation with family presence (40%), followed by communication training with stressors (20%). The trainings primarily occurred in controlled simulated environments (50%). In 90% of the cases, there was immediate debriefing, and the majority presented a high level of fidelity (70%), with actors representing family members and mannequins simulated as patients. For a better understanding, Figure 2 was developed.

*WOS - Web of Science; **UTI - Pediatric Intensive Care Unit; APH - Pre-Hospital Care; SME - Emergency Medical Service.

In the process of synthesizing the studies, variables such as reference, author, publication year, country, language and platform, design, population, context, educational intervention, and skills were organized in Chart 3.

**Chart 3:** Synthesis of the studies analyzed regarding the incorporation of the family approach in clinical simulation training for first responders, in the pediatric emergency context (n = 10), 2024

Code/ Reference	Author/Year	Country/ Language/ Platform	Design	Population	Context	Educational Intervention and Skills
E_1_^(29)^	Pye; Kane; Jones, 2010	USA/English MEDLINE	Preand post-test research	Nurses	Pediatric ICU	Educational program with clinical simulations of resuscitation for congenital heart disease, including family presence at the bedside.
E_2_^(30)^	Fisher *et al,* 2014	USA/English MEDLINE	Mixed Experimental	Nurses	Pediatric Hospital	Validated clinical simulation session for communication training in stressful situations.
E_3_^(31)^	Ayub *et al*, 2016	USA/English MEDLINE	Cross-sectional Qualitative	First Responders and Health Professionals	Pre-Hospital Care and Emergency Medical Service	Technical training with family involvement for pre-hospital emergency providers.
E_4_^(32)^	Sweeney *et al*, 2020	Ireland/ English Embase	Experimental with Focus Group Technique	Residents	Pediatric Hospital	Training course with clinical simulation for communication in challenging pediatric situations such as death and pediatric emergencies.
E_5_^(33)^	Deacon *et al,* 2020	Canada/English MEDLINE	Multicenter Qualitative Study	Health Professionals	Pediatric Hospital	Educational interventions with recorded simulated events on resuscitation to manage family presence during care.
E_6_^(34)^	Schafer; Kremer, 2022	USA/English WOS	Systematic Review	Health Professionals	-	Analysis of studies with clinical simulation scenarios involving family presence during resuscitation, aimed at training affective communication skills.
E_7_^(35)^	Lewis *et al,* 2022	USA/English MEDLINE	Technology Development and Application	Residents and Health Professionals	Emergency Medical Service	The educational program includes interactive activities with clinical simulation, with family presence during child resuscitation, as well as workshops on family engagement and communication techniques.
E_8_^(36)^	Bordessoule *et al,* 2022	Switzerland/ English MEDLINE	Observational	Health Professionals	Pediatric ICU	Program of simulations with clinical scenarios for training communication with the family in stressful situations.
E_9_^(37)^	Bush; Woodley, 2022	USA/English MEDLINE	Preand post-intervention Experimental	Nurses	Pediatric ICU	Structured educational session with content presentation, discussion, simulation videos, and parent testimonials to enhance professionals' confidence in emergency care in the presence of the family.
E_10_^(38)^	Cooper *et al,* 2024	Australia/ English MEDLINE	Mixed Quasi-experimental Preand Post-Intervention	Health Professionals	Pediatric Hospital	Online educational package with clinical simulation for technical training associated with family involvement.

*Notes: USA - United States of America; Ped. ICU - Pediatric Intensive Care Unit; APH - Pre-Hospital Care; EMS - Emergency Medical Service; WOS - Web of Science.*

The review underscored the growing importance of recognizing family presence during pediatric emergency care, although first responders continue to prioritize patient care^(31)^. After simulations, first responders demonstrated a significant increase in confidence regarding family involvement^(32)^, influenced by the resuscitation environment and emotional and behavioral dynamics^(33)^. Improvements in clinical and practical skills^(34)^ led to reduced emotional stress among the teams^(36)^. The post-intervention outcomes were favorable, showing that integrating multiple clinical scenarios enhances participants’ learning^(38)^ and improves professional conduct.

## DISCUSSION

Patient and Family Centered Care (PFCC) is a healthcare philosophy that plays a crucial role in healthcare delivery by recognizing the family as a partner in care and decision-making^(39)^. To ensure the success of this approach, it is crucial that healthcare professionals are aware and committed^(39)^. Analysis of the studies shows that simulation practice in pediatric emergency situations, involving first responders and family presence, has significantly impacted both the performance of healthcare professionals and health outcomes for patients and families^(29-38)^.

Research exploration revealed that the United States stood out as the country with the highest volume of publications^(29-31, 34,35,37)^. The concept of PFCC was formalized in 1992^(1,40)^ by the Family-Centered Care Institute, based in the USA^(1,40)^, confirming that developed countries were the first to promote the development of PFCC^(40)^. The first recorded publication on clinical simulation training in pediatric emergency contexts was found in the Medline database, dated 2010^(29)^. Since then, the volume of publications has progressively increased, peaking in 2022^(34-37)^.

Given the clear benefits of this philosophy, its adoption has become widely accepted across various areas of pediatric healthcare^(40)^. This facilitated the implementation of simulation training in various units, initially in pediatric and neonatal intensive care units^(29,36,37)^, pediatric cardiology programs^(29)^, emergency rooms^(31,32,35)^, pediatric clinical units^(30,32,33,38)^, and to a lesser extent, in the pre-hospital contexto^(31)^, with a single study associated with a hospital unit for emergency pediatric simulation with first responders. It is important to note that the joint 2006 policy guidelines from the American Academy of Pediatrics and the American College of Emergency Physicians^(36)^, as well as the 2015 European Resuscitation Council Guidelines for Resuscitation, promote family presence during resuscitation attempts^(41)^.

In this context, this review emphasizes the growing multi-professional participation in trainings over the years^(31-36,38)^. Although nurses are generally more present and supportive of PFCC, many lack the knowledge and self-confidence necessary to implement it effectively^(30,42)^. Transitioning to a family-centered approach faces cultural challenges and perceptions of care, requiring adaptation and acceptance for successful implementation, even though nurses’ self-efficacy does not yet indicate full readiness for this transformation^(42)^. This is also reflected in the approach of first responders who, in critical pediatric events, prioritize patient care over the family approach^(31)^.

Evidence indicates significant progress in the use of simulations in health education^(29-38)^. Among various strategies, this review demonstrates that high-fidelity simulation is increasingly being employed^(29,31-34,36-38)^, enhancing clinical reasoning and aiding in decision-making in complex cases^(43)^.

The studies highlight the importance of developing communication skills and emotional support in pediatric emergency care^(29,30,32,34,36,38)^, particularly in resuscitation scenarios^(29,32-35,37)^. The quality of both verbal and non-verbal communication is essential for families, as their active involvement can mitigate feelings of helplessness and counteract traumatic grief^(9)^. While the use of simulations in healthcare has sparked interest in developing non-technical competencies such as communication, these are still less studied than technical competencies, with variable outcomes^(42)^. Managing quality care and safety in emergencies requires communication skills to build trust and achieve goals^(42)^. The effective performance of these skills can overcome environmental and workforce barriers and is crucial for patientand family-centered care^(31)^.

The limitations identified in this review relate to the nature of the clinical simulation intervention. These include distinguishing between the controlled simulation setting and the real environment^(31)^, cultural barriers among teams^(31)^, structural problems in simulations, unvalidated scenarios^(34)^, actors with inconsistent performances or known faces^(31,33)^, prolonged duration of simulations^(35)^, and the absence of part of the target population during the stages^(35,37)^.

Finally, the presence of families during pediatric emergency simulations not only reduces stress for healthcare professionals but also enhances their responses, underscoring the importance of simulation-based health education. In addition to increasing team confidence and identifying knowledge gaps, the integration of simulation, communication, and emotional support is crucial for enhancing the quality of care and improving the family experience. Investments in these aspects promote a more empathetic and patient-centered care environment, reinforcing the need to advance research in this context in other settings.

### Study Limitations

Most records identified through the PCC acronym^(23)^ described technical studies on clinical simulation training in pediatric emergencies for first responders, due to the difficulty of finding specific descriptors for PFCC. This limitation may have constrained the searches, as most articles used “family-centered care” and “presence of family” as keywords. Additionally, using the descriptor “emergency responders” resulted in unfruitful searches, necessitating the inclusion of “health personnel” to access studies related to healthcare professionals in hospital and pre-hospital settings. The predominance of studies conducted in the United States, where this philosophy originated, also narrows the scope of discussions with other realities.

### Contributions to research and health fields

The research emphasizes clinical simulation as an indispensable tool for training in pediatric emergencies with family involvement. Evidence highlights benefits such as increased self-confidence among professionals, adoption of effective tactics to reduce team stress, and improved communication with families, especially in hospital environments.

It is noteworthy that promoting further research in this field can expand knowledge and enhance technical skills in pediatric emergencies with family presence, providing training and support for professionals. Extending family-centered clinical simulation strategies to various countries could significantly impact the promotion of humane pediatric emergency care globally.

## CONCLUSIONS

The evidence from this research demonstrates that simulations are effective in overcoming barriers in interactions with family members of children during emergency care, allowing professionals to gain confidence and enhance technical and communication skills in a controlled environment. However, family interaction remains secondary in pediatric emergency training, and there is a need for greater emphasis.

Family-centered pediatric emergency care ensures comprehensive and safe assistance, overcoming challenges in critical situations. Future training and research initiatives should focus on including families, adopting a more holistic and collaborative approach to patient care.
