# Supplementary material for: Clinical simulation training for first responders in pediatric emergencies with family interaction: a scoping review
Source: Rev Bras Enferm. 2025 Jan 13;78(1):e20240218. doi: 10.1590/0034-7167-2024-0218 (PMC11731850; doi:10.1590/0034-7167-2024-0218)
Supplement: Translation

# Translation: Treinamentos por simulação clínica para socorristas na emergência pediátrica com interação familiar: revisão de escopo

**DOI:** 10.1590/0034-7167-2024-0218pt

**Authors:** Karine Silva Fogaça, Gabriella Figueiredo Marti, Andréia Insabralde de Queiroz Cardoso, Rodrigo Guimarães dos Santos Almeida, Fernanda Ribeiro Baptista Marques, Maria Angélica Marcheti

**Affiliations:** I Universidade Federal de Mato Grosso do Sul. Campo Grande, Mato Grosso do Sul, Brasil

**Keywords:** Socorristas, Profissionais de Saúde, Treinamento por Simulação, Criança, Enfermagem Familiar

## Abstract

**Objetivo:**

Mapear estudos sobre treinamentos por simulação clínica direcionados aos socorristas durante emergências com crianças, focados na interação com as famílias.

**Métodos:**

Revisão de escopo fundamentada conforme diretrizes do referencial *JBI Manual for Evidence Syntheses* e relatada conforme o checklist PRISMA-ScR, em oito bases de dados e literatura cinzenta, sem recorte temporal ou idiomático.

**Resultados:**

Dez estudos selecionados indicaram que as publicações são majoritariamente dos Estados Unidos. Predominaram os treinamentos por simulação conduzidos em ambientes hospitalares, com apenas um estudo associado ao contexto pré-hospitalar. Os principais designs utilizados envolvem a temática de reanimação pediátrica e cenários em ambientes simulados de alta fidelidade.

**Conclusão:**

Os treinamentos de simulação são eficazes, aumentam a confiança dos profissionais e aprimoram a comunicação com as famílias. Contudo, a concentração em países desenvolvidos e hospitais ressalta a necessidade de capacitação em outras ambiências, como o atendimento pré-hospitalar, para integração do contexto técnico com a abordagem familiar.

## INTRODUÇÃO

Profissionais que envolvem a família no plano de cuidados, independentemente do estado de saúde, guiam-se pelos princípios do Cuidado Centrado no Paciente e na Família (CCPF)^(1)^. Esse modelo de cuidado preconiza uma assistência clara e objetiva, com planejamento, prestação e avaliação integrados, visando ao bem-estar de indivíduos e famílias^(2)^. O CCPF tornou-se ideal para profissionais que atuam na assistência a pacientes e suas famílias nos diversos serviços de saúde^(3)^.

A família é considerada parte essencial do processo de saúde-doença de seus membros^(4)^. Durante atendimentos emergenciais, a presença junto ao paciente, conhecida como presença à beira do leito, é valorizada^(5-8)^. Estudos destacam os benefícios do envolvimento da família no cuidado pediátrico, que incluem segurança, redução da ansiedade, apoio emocional, maior compreensão e satisfação com a assistência. Além disso, a presença familiar no momento da morte auxilia no processo de luto^(7-9)^.

A filosofia do CCPF está historicamente ligada ao atendimento de crianças e ao contexto hospitalar, principalmente por ter se originado em unidades de internação pediátrica^(10)^. Vale ressaltar que, em condições de emergência, a família se encontra vulnerável, sob estresse e sofrimento. Quando em domicílio, os familiares geralmente estão presentes desde o surgimento dos primeiros sintomas de gravidade até a chegada dos socorristas. No contexto dinâmico do atendimento pré-hospitalar (APH), existem limitações à presença dos familiares. Os profissionais de saúde buscam interação com a família principalmente para obter informações sobre o paciente; no entanto, é comum que o feedback não seja transmitido de forma clara. Nesse cenário, a abordagem técnica assume uma importância fundamental na entrega dos cuidados, sendo que a urgência das tarefas impacta diretamente sua eficácia^(11)^.

Situações emergenciais pediátricas demandam atenção especial dos profissionais de saúde devido às peculiaridades biológicas e psicológicas das crianças, exigindo recursos especializados^(3)^. Os profissionais devem estar atualizados nos padrões de avaliação e tratamento, bem como familiarizados com dispositivos pediátricos, para evitar adversidades^(12)^. Assim, os cursos de capacitação profissional geralmente focam em aspectos técnicos, o que limita o desenvolvimento de habilidades de comunicação e empatia com a família^(13)^.

Implementar o modelo centrado na família no atendimento de emergência pode ser desafiador^(14)^, mas permitir que as famílias escolham permanecer ao lado do paciente é uma forma de introduzi-lo^(15-17)^. Para isso, é necessário aprimorar a qualificação e capacitação dos socorristas, promovendo o aprendizado contínuo e uma abordagem reflexiva, com vistas a uma assistência integral e de qualidade^(18)^.

Para a capacitação desses profissionais, o uso de cenários simulados tem se mostrado eficaz, reduzindo erros e promovendo segurança e qualidade na aprendizagem^(19)^. A simulação clínica é uma abordagem motivadora que melhora a adesão e reduz a evasão em processos educacionais, além de avaliar competências técnicas e não técnicas, como comunicação e profissionalismo^(20,21)^, evidenciando resultados positivos no aperfeiçoamento e satisfação dos profissionais de saúde^(22)^. No entanto, ainda há poucos treinamentos em emergências pediátricas com foco na atenção centrada na família.

Para este estudo, foram conduzidas buscas preliminares nas bases de dados *JBI Evidence Synthesis, PROSPERO Systematic Review, Open Science Framework* (OSF) e *Cochrane Database of Systematic Reviews* (CDSR). Essas buscas não identificaram revisões sistemáticas ou de escopo concluídas ou em andamento relacionadas à temática.

Diante do exposto, é crucial reconhecer as ações focadas na interação familiar durante os treinamentos de simulação clínica para socorristas em emergências pediátricas. Tal análise pode fomentar iniciativas de capacitação profissional e auxiliar na criação de normas institucionais que garantam e incentivem a presença da família durante o atendimento de emergência pediátrica.

## OBJETIVO

Mapear estudos sobre treinamentos por simulação clínica, direcionados aos socorristas durante emergências em crianças, com foco na interação com as famílias.

## MÉTODOS

### Aspectos éticos

Não foi necessária a apreciação pelo Comitê de Ética em Pesquisa com Seres Humanos, visto que o material utilizado é de domínio público e não envolveu seres humanos.

### Tipo de estudo

Trata-se de uma revisão de escopo, estruturada conforme as diretrizes do *JBI Manual for Evidence Syntheses^(^* ^23)^. Esse tipo de técnica é conveniente para o levantamento de evidências emergentes, visando desenvolver um “mapa conceitual”, fornecer uma visão geral e ampla de uma questão e identificar lacunas na base de conhecimento da pesquisa, orientando a necessidade de novas investigações^(24,25)^. Para conduzir os passos desta revisão de escopo, adotaram-se as cinco etapas recomendadas pelo JBI, a saber: 1) Identificação da pergunta da pesquisa; 2) Busca dos estudos relevantes; 3) Seleção dos estudos; 4) Mapeamento dos dados; 5) Agrupamento, resumo e apresentação dos resultados.

Para o relato desta revisão, foi utilizado o checklist segundo as recomendações do *Preferred Reporting Items for Systematic reviews and Meta-Analyses extension for Scoping Reviews* (PRISMA-ScR)^(26)^.

### Procedimento metodológico

Os objetivos, critérios de inclusão e métodos para esta revisão de escopo foram definidos previamente e registrados em um protocolo de revisão de escopo elaborado conforme as diretrizes do JBI^(23)^. O protocolo foi registrado na plataforma *Open Science Framework* (OSF) sob o DOI: https://doi.org/10.17605/OSF.IO/YR9QA ^(27)^.

O objetivo do estudo, a questão norteadora e a estratégia de busca foram definidos conforme o mnemônico PCC^(23)^, detalhados no Quadro 1. A questão orientadora desenvolvida para este estudo foi: Como tem sido o processo de interação familiar nos treinamentos de simulação clínica para socorristas no contexto emergencial pediátrico?

**Quadro 1:** Mnemônico e descritores utilizados nas buscas, 2024

Mnemônico	Descritores	DeCS/MeSH/EMTREE
**População:** Socorristas	*Emergency Responders* *Health Personnel*	Socorristas *Emergency Responders* *Emergency First Responder* *First Responder* Pessoal de Saúde *Health Personnel* *Health Care Provider* *Healthcare Provider* *Healthcare Worker* *Health Care Professional*
**Conceito:** Interação familiar nos treinamentos de simulação clínica	*Simulation Training*	Treinamento por Simulação *Simulation Training* *Interactive Learning*
**Contexto:** Contexto emergencial pediátrico.	*Child*	Criança *Child* *Children*

Os critérios de inclusão selecionaram evidências de estudos que utilizaram treinamentos por simulação clínica como estratégia educacional voltada para profissionais de saúde ou socorristas no contexto de emergência pediátrica. Foram considerados artigos completos, documentos de sociedades, manuais, *guidelines* e protocolos, documentos governamentais, teses e dissertações disponíveis na íntegra que abordassem a temática. Além disso, as listas de referências das publicações foram revisadas para que todos os estudos relativos ao tema de interesse fossem identificados. Vale ressaltar que não houve recorte temporal ou limitação de idioma. Foram excluídos do estudo monografias, resenhas, editoriais, cartas ao editor, opiniões de especialistas, resumos, anais e correspondências.

### Estratégia de busca

A estratégia de busca foi organizada por dois pesquisadores e validada por outro pesquisador com experiência neste processo. A partir da questão de pesquisa, foram selecionados descritores controlados e indexados do *Medical Subject Headings* (MeSH), *EMBASE Subject Headings* (EMTREE), *CINAHL Headings* e Descritores em Ciências da Saúde (DeCS). As palavras-chave foram selecionadas a partir de sugestões dos vocabulários controlados e de leitura prévia minuciosa acerca da temática. Para a combinação desses descritores, foram utilizados os operadores booleanos OR e AND, conforme as particularidades de cada base de dados, conforme mostrado no Quadro 2.

**Quadro 2:** Estratégias de busca em bases de dados, 2024

Base de dados	Estratégias de Busca
*Embase* (*Elsevier*)	*(‘rescue personnel’/exp OR ‘rescue personnel’ OR ‘emergency responders’ OR ‘emergency first responder’ OR ‘first responder’ OR ‘health care personnel’/exp OR ‘health care personnel’ OR ‘health personnel’ OR ‘health care provider’ OR ‘healthcare provider’ OR ‘healthcare worker’ OR ‘health care professional’) AND (‘simulation training’/exp OR ‘simulation training’ OR ‘interactive learning’/exp OR ‘interactive learning’) AND (‘child’/exp OR ‘child’ OR ‘children’) AND [embase]/lim*
Biblioteca Virtual em Saúde/BVS	*((emergency responders) OR (emergency first responder) OR (first responder)) OR ((health personnel) OR (health care provider) OR (healthcare provider) OR (healthcare worker) OR (health care professional)) AND ((simulation training) OR (interactive learning)) AND ((child) OR (children))*
MEDLINE Pubmed	*(“Emergency Responders”[MeSH Terms] OR “Emergency First Responder”[Title/Abstract] OR “First Responder”[Title/Abstract] OR (“Health Personnel”[MeSH Terms] OR “Health Care Provider”[Title/Abstract] OR “Healthcare Provider”[Title/Abstract] OR “Healthcare Worker”[Title/Abstract] OR “Health Care Professional”[Title/Abstract])) AND (“Simulation Training”[MeSH Terms] OR “Interactive Learning”[Title/Abstract]) AND (“Child”[MeSH Terms] OR “Children”[Title/Abstract])*
*Web of Science* *WOS*	*(((ALL=(“Emergency Responders” OR “Emergency First Responder” OR “First Responder”)) OR ALL=(“Health Personnel” OR “Health Care Provider” OR “Healthcare Provider” OR “Healthcare Worker” OR “Health Care Professional”)) AND ALL=(“Simulation Training” OR “Interactive Learning”)) AND ALL=(“Child” OR “Children”)*
*Scopus (Elsevier)*,	*(ALL(‘emergency AND responders’ OR ‘emergency AND first AND responder’ OR ‘first AND responder’) AND ALL(‘simulation AND training’ OR ‘interactive AND learning’) AND ALL(‘child’ OR ‘children’))*
*Cochrane Library*	*((emergency responders) OR (emergency first responder) OR (first responder)):ti,ab,kw OR ((health personnel) OR (health care provider) OR (healthcare provider) OR (healthcare worker) OR (health care professional)):ti,ab,kw AND ((simulation training) OR (interactive learning)):ti,ab,kw AND ((child) OR (children)):ti,ab,kw*
CINAHL EBSCOhost *with Full Text*	*( MH ”Emergency Responders” OR “Emergency Responders” OR “Emergency First Responder” OR “First Responder” ) OR ( MH ”Health Personnel” OR “Health Personnel” OR “Health Care Provider” OR “Healthcare Provider” OR “Healthcare Worker” OR “Health Care Professional” ) AND ( MH “Simulation” OR “Simulation Training” OR “Interactive Learning” ) AND ( MH “Chid” OR “Child” OR “Children” )*
*ScienceDirect*	*{emergency responders} or {health personnel} and {simulation training} and {child}*

Foi realizada uma busca prévia na base de dados *National Library of Medicine* (MEDLINE/PubMed) para verificar a viabilidade da pesquisa. As palavras-chave foram definidas com a utilização do MeSH. As buscas das publicações ocorreram entre janeiro e março de 2024, por meio do acesso proxy da Universidade Federal de Mato Grosso do Sul (UFMS), a partir do registro no portal de periódicos da Coordenação de Aperfeiçoamento de Pessoal de Nível Superior (CAPES), via Comunidade Acadêmica Federada (CAFe).

As bases de dados consultadas incluíram: *Embase* (Elsevier), BVS (Biblioteca Virtual em Saúde), *National Library of Medicine* (MEDLINE/PubMed), *Web of Science Core Collection* (Clarivate Analytics), *Scopus* (Elsevier), *Cochrane Library, CINAHL EBSCOhost with Full Text* e *ScienceDirect*.

Além disso, foi realizada uma busca em fontes não indexadas, conhecidas como literatura cinzenta, utilizando as mesmas palavras-chave, combinadas com os operadores booleanos OR e AND em inglês e seus equivalentes em português. As fontes consultadas incluíram o Catálogo de Teses e Dissertações da Coordenação de Aperfeiçoamento de Pessoal de Nível Superior (CAPES), a Biblioteca Digital de Teses e Dissertações (BDTD), Banco Internacional de Acesso Aberto a Teses e Dissertações (OATD), *Academic Archive Online* (DIVA), Repositório Científico de Acesso Aberto de Portugal (RCAAP) e Sistema de Informação *Open Gray*.

Os estudos foram exportados para o gerenciador de referências Mendeley para identificação e exclusão de duplicatas. Para seleção e avaliação em relação aos critérios de inclusão, os artigos foram migrados para o software Rayyan^(28)^. A pré-seleção dos dados foi realizada pela leitura do título e do resumo por dois revisores, de forma independente. Não houve discordância entre os revisores, portanto, não foi necessário incluir um terceiro pesquisador ou contatar os autores primários sobre os dados.

O refinamento dos estudos foi fundamentado conforme os critérios de elegibilidade. Foram incluídos os artigos que continham os três elementos da mnemônica PCC^(23)^ associados à abordagem com a presença familiar, sem limite de idioma e tempo de publicação. Estudos que não responderam à questão norteadora da pesquisa e que não incluíram a presença da família no contexto emergencial foram excluídos.

Os estudos que compuseram a amostra final para análise foram então lidos na íntegra, com a coleta de dados realizada em planilha estruturada no software Microsoft Excel, contendo as seguintes variáveis de análise: citação, ano de publicação, origem, idioma, delineamento, população, contexto, intervenção educativa, treinamentos de simulação, objetivos do estudo, resultados e limitações.

Os dados foram descritos e apresentados por meio de figuras e quadros explicativos, com análise descritiva dos achados.

## RESULTADOS

Inicialmente, foram identificadas 2.538 produções, das quais 2.467 em bases de dados e 71 em outras fontes. Após a exclusão de 141 duplicatas, restaram 2.397 registros. Na primeira seleção, que envolveu a leitura de títulos e resumos, 2.375 registros foram excluídos por não atenderem aos critérios de inclusão, resultando em 22 estudos para análise. Em uma nova triagem, cinco estudos foram excluídos por não abordarem a interação familiar, três por divergirem do conceito adotado nesta revisão, um por apresentar contexto diferente do definido, e dois por não estarem disponíveis na íntegra. Ao final, a revisão foi composta por 10 publicações obtidas nas bases de dados, conforme demonstrado no fluxograma PRISMA-ScR^(26)^ (Figura 1).

**Figura 1:** Fluxograma PRISMA-ScR de identificação e seleção de estudos em base de dados, 2024

O período de publicação dos estudos elegíveis variou entre os anos de 2010 e 2024, sem delimitação de marco temporal, com maior ocorrência em 2022 (40%). Os estudos foram predominantemente dos Estados Unidos (60%), seguidos por Irlanda, Canadá, Suíça e Austrália (10% cada). A maioria dos estudos (80%) foi oriunda da *National Library of Medicine* (MEDLINE/PubMed).

As intervenções com simulações clínicas envolveram principalmente treinamentos multiprofissionais com médicos, enfermeiros e fisioterapeutas (60%). Enfermeiros em treinamento exclusivo apresentaram uma participação significativa, com 30%. Vale ressaltar a integração do enfermeiro com a equipe multiprofissional, demonstrando-se como a categoria profissional com maior frequência na realização de treinamentos de simulação clínica, com a presença da família durante emergências pediátricas (90%).

Todos os estudos foram conduzidos em hospitais, sendo que 10% também estavam associados a outras ambiências, mas nenhum exclusivamente em cenários de APH. Quanto ao design dos cenários simulados realizados, os estudos mostraram uma predominância da temática de reanimação pediátrica com a presença da família (40%), seguida por treinamentos de comunicação com estressores (20%). Os treinamentos ocorreram majoritariamente em ambientes simulados controlados (50%). Em 90% dos casos, houve *debriefing* imediato, e a maioria apresentou alto nível de fidelidade (70%), com atores representando familiares e manequins simulados representando pacientes. Para melhor compreensão, foi desenvolvida a Figura 2.

**Figura 2:** Desfecho e *design* dos treinamentos de simulação clínica com abordagem familiar, 2024

*WOS - Web of Science; **UTI - Unidade de Terapia Intensiva Pediátrica; APH - Atendimento pré-hospitalar; SME - Serviço Médico de Emergência.

No processo de síntese dos estudos, foram organizadas variáveis como referência, autor e ano de publicação, país, idioma e plataforma, delineamento, população, contexto, intervenção educativa e habilidades no Quadro 3.

**Quadro 3:** Síntese dos estudos analisados referente a inserção da abordagem familiar nos treinamentos de simulação clínica para socorristas, no contexto emergencial pediátrico (n = 10), 2024

Código/ Referência	Autor/Ano	País/Idioma Plataforma	Delineamento	População	Contexto	Intervenção Educativa e Habilidades
E_1_^(29)^	Pye; Kane; Jones, 2010	EUA/Inglês MEDLINE	Pesquisa pré e pós-teste	Enfermeiros	UTI Ped	Programa educacional com simulações clínicas de reanimação em doença cardíaca congênita, incluindo a presença da família à beira do leito.
E_2_^(30)^	Fisher *et al,* 2014	EUA/Inglês MEDLINE	Experimental Misto	Enfermeiros	Hospital Pediátrico	Sessão validada de simulação clínica para treinamento em comunicação em situações de estresse.
E_3_^(31)^	Ayub *et al*, 2016	EUA/Inglês MEDLINE	Qualitativo Transversal	Socorristas e Profissionais de Saúde	APH e SME	Treinamento técnico com a inserção familiar para provedores de emergência pré-hospitalares.
E_4_^(32)^	Sweeney *et al*, 2020	Irlanda/ Inglês Embase	Experimental com técnica de Grupo Focal	Residentes	Hospital Pediátrico	Curso de formação com simulação clínica para comunicação em situações desafiadoras na pediatria como morte e emergências pediátricas.
E_5_^(33)^	Deacon *et al,* 2020	Canadá/Inglês MEDLINE	Estudo Qualitativo multicêntrico	Profissionais de saúde	Hospital Pediátrico	Intervenções educativas com eventos simulados gravados sobre reanimação para gerenciamento da presença de família durante o atendimento.
E_6_^(34)^	Schafer; Kremer, 2022	EUA/Inglês *WOS*	Revisão Sistemática	Profissionais de saúde	-	Análise de estudos com cenários de simulação clínica envolvendo a presença da família durante a reanimação, visando o treinamento de competências afetivas de comunicação.
E_7_^(35)^	Lewis *et al,* 2022	EUA/Inglês MEDLINE	Desenvolvimento de tecnologia e aplicação	Residentes e Profissionais de saúde	SME	O programa educacional inclui atividades interativas com simulação clínica, com a presença da família durante a reanimação da criança, além de workshops sobre técnicas de envolvimento e comunicação com a família.
E_8_^(36)^	Bordessoule *et al,* 2022	Suíça/Inglês MEDLINE	Observacional Experimental pré e pós-intervenção	Profissionais de saúde	UTI Ped	Programa de simulações com cenários clínicos para treinamento da comunicação com a família em situações de estresse.
E_9_^(37)^	Bush; Woodley, 2022	EUA/Inglês MEDLINE	Experimental pré e pós-intervenção	Enfermeiros	UTI Ped	Sessão educativa estruturada com apresentação do conteúdo, discussão, vídeos de simulação e depoimento dos pais para aumentar a confiança dos profissionais frente ao atendimento emergencial na presença da família.
E_10_^(38)^	Cooper *et al,* 2024	Austrália/Inglês MEDLINE	Misto Quase-experimental pré e pós-intervenção	Profissionais de saúde	Hospital Pediátrico	Pacote educacional *on-line* com simulação clínica para treinamento técnico associado a inserção familiar.

*Notas: EUA - Estados Unidos da América; UTI Ped. - Unidade de Terapia Intensiva Pediátrica; APH - Atendimento pré-hospitalar; SME - Serviço Médico de Emergência; WOS - Web of Science.*

Os resultados da revisão destacaram a importância crescente do reconhecimento da presença da família durante os atendimentos de emergência pediátrica, embora os socorristas mantenham a prioridade na atenção ao paciente^(31)^. Após simulações, os socorristas demonstraram aumento significativo na confiança para envolver as famílias^(32)^, influenciados pelo ambiente de reanimação e dinâmicas emocionais e comportamentais^(33)^. As melhorias nas competências clínicas e práticas^(34)^ resultaram na redução do estresse emocional nas equipes^(36)^. Os resultados pós-intervenção foram positivos, evidenciando que a integração de múltiplos cenários clínicos beneficia o aprendizado dos participantes^(38)^ e melhora as condutas profissionais.

## DISCUSSÃO

O CCPF é uma filosofia de saúde que desempenha um papel relevante na prestação de cuidados de saúde, uma vez que reconhece a família como parceira nos cuidados e na tomada de decisões^(39)^. Para garantir o êxito dessa abordagem, é crucial que os profissionais de saúde estejam conscientes e comprometidos^(39)^. Ao analisar os estudos, verifica-se que a prática de simulação em situações de emergências pediátricas para socorristas, aliada à presença de familiares, tem revelado impactos substanciais tanto no desempenho dos profissionais de saúde quanto nos resultados em saúde dos pacientes e das famílias^(29-38)^.

Ao explorar as pesquisas, constatou-se que os Estados Unidos se destacaram como o país com o maior volume de publicações^(29-31,34,35,37)^. O conceito de CCPF foi formalizado em 1992^(1,40)^ pelo *Family-Centered Care Institute*, sediado nos EUA^(1,40)^, o que corrobora que os países desenvolvidos foram os primeiros a promover o desenvolvimento do CCPF^(40)^. A primeira publicação registrada sobre treinamentos de simulação clínica em contexto emergencial pediátrico foi encontrada na base de dados Medline, datada de 2010^(29)^. Desde então, o volume de publicações apresenta um aumento progressivo, culminando em seu ápice em 2022^(34-37)^.

Devido aos benefícios claros dessa filosofia, sua adoção se tornou amplamente aceita em várias áreas de cuidados de saúde pediátrica^(40)^. Isso abriu caminho para a implementação de treinamentos de simulação em diversas unidades, inicialmente em unidades de cuidados intensivos pediátricos e neonatais^(29,36,37)^, programas de cardiologia pediátrica^(29)^, salas de emergência^(31,32,35)^, unidades clínicas pediátricas^(30,32,33,38)^ e, em menor número, no contexto pré-hospitalar^(31)^, com um único estudo associado a uma unidade hospitalar para a simulação em emergência pediátrica com socorristas. Vale ressaltar que as orientações da política conjunta de 2006 da Academia Americana de Pediatria e do Colégio Americano de Médicos de Emergência^(36)^, bem como as Diretrizes do Conselho Europeu de Reanimação para Reanimação de 2015, promovem a presença da família durante tentativas de reanimação^(41)^.

Associado a esse contexto, esta revisão destaca a crescente participação multiprofissional nos treinamentos ao longo dos anos^(31-36,38)^. Embora os enfermeiros estejam geralmente mais presentes e dispostos a apoiar o CCPF, muitos carecem do conhecimento e da autoconfiança necessários para implementá-lo eficazmente^(30,42)^. A transição para uma abordagem centrada na família enfrenta desafios culturais e de percepção do cuidado, com a necessidade de adaptação e aceitação para uma implementação bem-sucedida, mesmo que a autoeficácia dos enfermeiros ainda não indique uma preparação completa para essa transformação^(42)^. Isso se reflete também no posicionamento dos socorristas, que, em eventos pediátricos críticos, priorizam o cuidado ao paciente em detrimento da abordagem familiar^(31)^.

As evidências indicam uma evolução significativa na utilização de simulações na área da educação em saúde^(29-38)^. Dentre as diversas estratégias, esta revisão demonstra que a simulação de alta fidelidade está sendo cada vez mais empregada^(29,31-34,36-38)^, favorecendo o raciocínio clínico e auxiliando na tomada de decisão em casos complexos^(43)^.

Os estudos destacaram a prioridade em desenvolver habilidades de comunicação e apoio emocional em atendimentos emergenciais pediátricos^(29,30,32,34,36,38)^, especialmente em cenários de reanimação^(29,32-35,37)^. A qualidade da comunicação, tanto verbal quanto não verbal, é fundamental para as famílias, pois seu envolvimento ativo pode neutralizar a experiência de desamparo e atuar contra o luto traumático^(9)^. Embora o uso de simulações em saúde tenha despertado interesse na formação de competências não técnicas, como a comunicação, essas ainda são menos estudadas que as competências técnicas, com resultados variáveis^(42)^. A gestão de cuidados de qualidade e segurança na emergência exige habilidades de comunicação para construir confiança e atingir objetivos^(42)^. O desempenho eficaz dessas habilidades pode superar barreiras ambientais e de mão de obra, sendo crucial para cuidados centrados no paciente e na família^(31)^.

As limitações identificadas nesta revisão estão relacionadas à natureza da intervenção por simulação clínica. Estas incluem a distinção entre o cenário controlado da simulação e o ambiente real^(31)^, barreiras culturais entre as equipes^(31)^, problemas estruturais nas simulações, cenários não validados^(34)^, atores com desempenho inconsistente ou conhecidos^(31,33)^, duração prolongada das simulações^(35)^ e ausência de parte da população-alvo durante as etapas^(35,37)^.

Por fim, a presença familiar durante simulações de emergência pediátrica não só reduz o estresse dos profissionais de saúde, mas também melhora suas respostas, evidenciando a importância da educação em saúde baseada em simulação. Além de aumentar a confiança da equipe e identificar lacunas de conhecimento, a integração entre simulação, comunicação e apoio emocional é crucial para aprimorar a qualidade dos cuidados e melhorar a experiência das famílias. Investimentos nesses aspectos promovem um ambiente de cuidados mais empático e centrado no paciente, reforçando a necessidade de promover pesquisas nesse contexto em outras ambiências.

### Limitações do estudo

A maioria dos registros identificados através do acrônimo PCC^(23)^ descrevia estudos técnicos sobre treinamento de simulação clínica em emergências pediátricas para socorristas, devido à dificuldade de encontrar descritores específicos para o CCPF. Essa condição pode ter limitado as buscas, visto que a maioria dos artigos utilizou “*family-centered care*” e “*presence of family*” como palavras-chave. Além disso, a utilização do descritor “*emergency responders*” resultou em pesquisas infrutíferas, sendo necessário acrescentar “*health personnel*” para alcançar estudos referentes aos profissionais de saúde em ambientes hospitalares e pré-hospitalares. A predominância de estudos realizados nos Estados Unidos, onde essa filosofia se originou, também restringe a amplitude das discussões com outras realidades.

### Contribuições para as Áreas de Pesquisa e Saúde

A pesquisa enfatiza a simulação clínica como uma ferramenta indispensável para treinamentos em emergências pediátricas com o envolvimento das famílias. As evidências apontam benefícios, como o aumento da autoconfiança dos profissionais, a adoção de táticas eficazes para reduzir o estresse da equipe e a melhoria da comunicação com os familiares, especialmente em ambientes hospitalares.

Vale ressaltar que promover pesquisas adicionais nesse campo pode ampliar o conhecimento e aprimorar habilidades técnicas em emergências pediátricas na presença da família, com capacitação e apoio aos profissionais. Ao expandir as estratégias de simulação clínica centradas na família para vários países, pode-se impactar significativamente a promoção do atendimento emergencial pediátrico humanizado em todo o mundo.

## CONCLUSÕES

As evidências desta pesquisa demonstraram que as simulações são eficazes para enfrentar barreiras na interação com familiares de crianças durante o atendimento emergencial, permitindo aos profissionais adquirir confiança e aprimorar habilidades técnicas e de comunicação em um ambiente controlado. No entanto, a interação familiar ainda é secundária em treinamentos de emergências pediátricas, havendo necessidade de maior ênfase.

Um atendimento emergencial pediátrico centrado na família assegura uma assistência integral e segura, superando desafios em situações críticas. Futuras iniciativas de treinamento e pesquisa devem focar na inclusão das famílias, com uma abordagem mais holística e colaborativa no cuidado ao paciente.
